# Clinical trials proposed for the VA Cooperative Studies Program: Success rates and factors impacting approval^☆^

**DOI:** 10.1016/j.conctc.2021.100811

**Published:** 2021-07-09

**Authors:** David R. Burnaska, Grant D. Huang, Timothy J. O'Leary

**Affiliations:** a Cooperative Studies Program, Office of Research and Development, Veterans Health Administration, Washington DC, 20420, USA; b Department of Pathology, University of Maryland School of Medicine, Baltimore, MD, USA

**Keywords:** Veterans, Peer review, Success rates, Letters of intent, Clinical trials

## Abstract

The process by which funding organizations select among the myriad number of proposals they receive is a matter of significant concern for researchers and the public alike. Despite an extensive literature on the topic of peer review and publications on criteria by which clinical investigations are reviewed, publications analyzing peer review and other processes leading to government funding decisions on large multi-site clinical trials proposals are sparse. To partially address this gap, we reviewed the outcomes of scientific and programmatic evaluation for all letters of intent (LOIs) received by the Department of Veterans Affairs (VA) Cooperative Studies Program (CSP) between July 4, 2008, and November 28, 2016. If accepted, these LOIs represented initial steps towards later full proposals that also underwent scientific peer review. Twenty-two of 87 LOIs were ultimately funded and executed as CSP projects, for an overall success rate of 25%. Most proposals which received a negative decision did so prior to submission of a full proposal. Common reasons for negative scientific review of LOIs included investigator inexperience, perceived lack of major scientific impact, lack of preliminary data and flawed or confused experimental design, while the most common reasons for negative reviews of final proposals included questions of scientific impact and issues of study design, including outcome measures, randomization, and stratification. Completed projects have been published in high impact clinical journals. Findings highlight several factors leading to successfully obtaining funding support for clinical trials. While our analysis is restricted to trials proposed for CSP, the similarities in review processes with those employed by the National Institutes of Health and the Patient Centered Outcomes Research Institute suggest the possibility that they may also be important in a broader context.

## 1 Background

The medical and prosthetic research program of the Department of Veterans Affairs (VA), though specifically dedicated to improving the health and well-being of Veterans, has nevertheless had a significant impact on the care of all Americans. The Cooperative Studies Program (CSP) carries out large-scale clinical trials and observational studies as part of this research mission [1,2]. Unlike the National Institutes of Health (NIH), which provides broad support to investigators through grants and cooperative agreements, the VA research program, and thus CSP, is an intramural program [3] that is, by law, carried out “in connection with the provision of medical care and treatment to veterans.” Nevertheless, broad participation from the academic community has, from the earliest days of CSP [4], come from collaborations with non-VA investigators and institutions, including the Department of Defense and the NIH.

Like NIH and other funding agencies, CSP relies on external scientific advisors to assist in the evaluation of investigations proposed for funding. Although some papers have examined the effectiveness of peer review for proposal selection [[5], [6], [7], [8]], they have for the most part not focused on clinical research. Both the Patient Centered Outcomes Research Institute (PCORI) and NIH have published policies for the evaluation of clinical research proposals [9,10]; PCORI has evaluated the use of its review criteria [11,12], but the extent to which this analysis extends to other funding agencies, such as VA or NIH, is uncertain. There are several differences between the processes employed by the VA CSP, those typically employed by NIH, and those used by other groups, including PCORI. For example, while CSP requires a two-step process, the use of the R34 Planning Grant mechanism is not required prior to submission of a full proposal to NIH. Like the R34, the CSP planning process that begins with approval for planning supports development of a research design, protocol finalization, and preparation of an operations manual, but does not generally support pilot or feasibility studies, and does not require submission of a planning budget, which is instead developed by the CSP Statistical Coordinating Center to whom an approved LOI is assigned for planning.

**Fig. 1:**
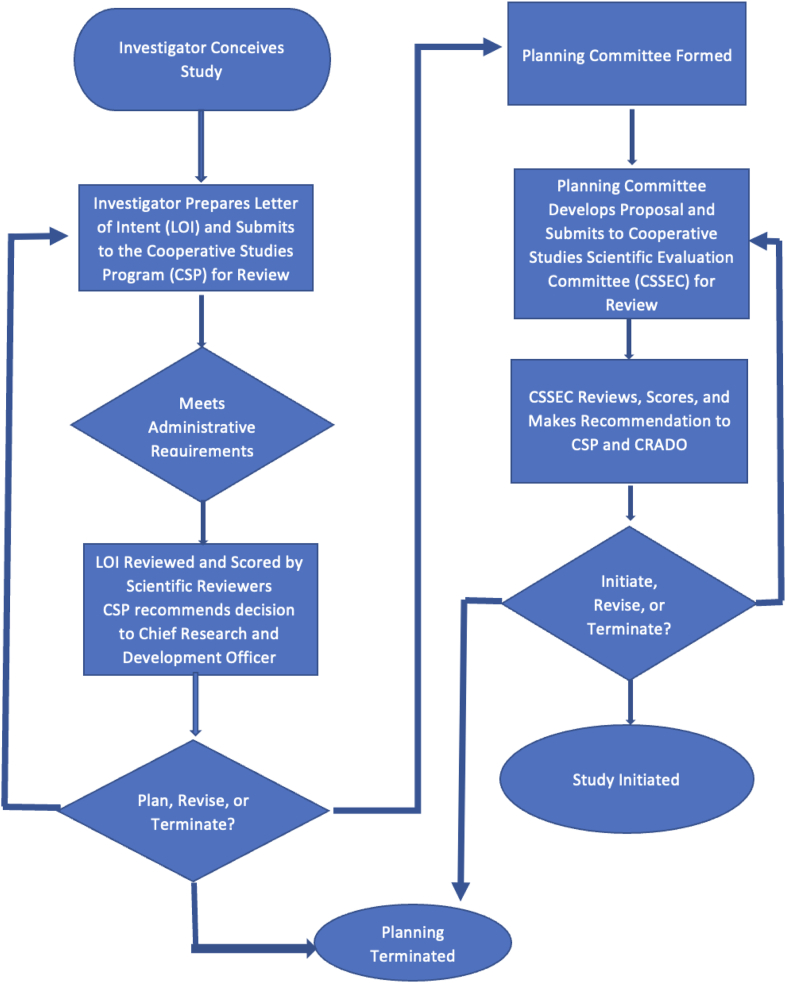
Flowchart outlining the basics of the VA Cooperative Studies Program proposal development and review process. As noted in the text, letters of intent that are flagged for revision or pre-planning may receive the assistance of one of the CSP Statistical Coordinating Centers as a part of the revision or approval process.

The processes used by CSP for proposal evaluation are described in detail at https://www.research.va.gov/programs/csp/update/guide.pdf, and utilize criteria that were, in many cases, identified in the earliest days of CSP [4]. Briefly, initial evaluation of trials proposed to CSP is conducted based on a structured letter of intent (LOI). An LOI is a detailed description of the proposed research and includes key details, an overview of the study objectives and design, relevance to the VA population and healthcare mission, potential impact of findings, and investigator experience. The LOI submission form is reproduced in the Supplemental Appendix. While conceptual in nature, the CSP LOI does require an investigator to demonstrate an important level of insight for a multi-site clinical trial that go beyond the scientific and clinical rationale for a study. Proposed design elements also ideally reflect a level of understanding for how a clinical question aimed at impacting the healthcare system can be rigorously answered. LOIs are reviewed by CSP for program relevance and to assure adherence to administrative requirements. Following this internal review, the LOI is sent to three or more scientific reviewers who are asked to make an initial evaluation of the importance, merit, and ethics of the proposed investigation and to score it on a scale of 1–5, where 5 is assigned to the most meritorious proposals. These external reviews are considered by program staff who recommend to the VA Chief Research and Development Officer (CRADO) whether support should be provided for a full project planning process, considering budgetary constraints and competing programmatic priorities. Fig. 1 depicts this two-stage process used by CSP. It is important to note that senior executive level approval not only reflects potential level of commitment of resources but also provides a higher level of ability for CSP to integrate with clinical and other priorities for the agency. This ability has been further advanced by a newer requirement for a healthcare system implementation plan to be established in partnership with the VA Quality Enhancement Research Initiative prior to study launch [13].

For those proposals which are approved for planning, support is provided by one of five CSP Statistical Coordinating Centers or one of four Epidemiology Centers, together with the CSP Pharmacy Coordinating Center (which also provides oversight of trial safety and represents VA as the sponsor for any Investigational New Drug or Investigational Device Exemption applications). Over a period of six months to one year the study proponents, other members of the study planning committee, and trialists or epidemiologists from the coordinating centers that include clinical, biostatistical, and quality expertise meet to create a finalized study design. Once the study has been designed, it is then submitted to the CSP Cooperative Studies Scientific Evaluation Committee (CSSEC), a chartered Federal Advisory Committee, which conducts a thorough and rigorous review of the final protocol, scoring it for scientific merit and making recommendations on funding. The CSSEC review is like that used by NIH, with one exception. Following initial discussion of written comments submitted before the meeting, the CSSEC invites the study proponent, study statistician, and coordinating center director associated with planning to discuss concerns raised by members of the CSSEC. There is thus an opportunity to clarify elements of the proposal “on the fly” and to respond to committee concerns. Following this, the committee determines whether to approve or disapprove the proposal, and, if approved, to score it on a scale of 10–50, where 10 represents the “best” score. Typically, scores between 10 and 22 are potentially “fundable,” although committee comments and concerns, availability of funds, and programmatic priority may all influence the final funding decisions made by the CRADO.

Although the CSSEC review is like that of NIH, the constitution of CSSEC is not made up of subspecialists with a primary focus on the subject matter of each proposal, but rather of a variety of academic medical faculty (including statistical faculty), with subspecialty expertise added to the panel using ad-hoc reviewers. This approach to constituting CSSEC is intended to assure that trials that are approved by CSSEC will be viewed as meaningful to a wide variety of medical professionals.

To the best of our knowledge, success rates and factors influencing success in proposing and ultimately executing CSP studies, or studies conducted by other organizations that use a similar LOI process, have not been previously reported. This paper addresses this gap.

## 2 Methods

All studies proposed to CSP between July 4, 2008, and November 28, 2016, for which an LOI was received, were evaluated; this timeframe reflects our ability to both easily retrieve records and to have sufficient time to allow resubmission of LOIs as well as submission, review, and rereview of final proposals, and completion of a few of those studies. The administrative decision on whether to seek scientific review was noted, and for those that were reviewed, the average score assigned by reviewers to the LOI was calculated, and the decisions on whether to proceed with the planning process were also recorded. A qualitative, and necessarily somewhat subjective, review of the comments made by the scientific reviewers was recorded to identify concerns specifically associated with investigator qualifications, quality of preliminary data supporting the study, knowledge of competing studies, concerns with subject recruitment, timelines, or statistical power, and clarity of proposed trial design.

For studies that underwent a planning process and were subsequently given a scientific evaluation by the CSSEC, average scores for both the initial submission and (if necessary) any resubmission were recorded, together with the decision whether to fund the proposal at that point. For proposals which were not funded, the reason for non-funding was identified by examining the decision letter. Concerns raised by the members of the CSSEC while evaluating the proposals were obtained from the CSSEC minutes and characterized in the same manner as those for LOIs.

Clinical trial LOIs for medical and surgical conditions were analyzed separately from those proposing non-randomized and/or observational (epidemiologic) studies and those involving mental health priorities, since administrative and agency/programmatic priority considerations differed among these three groups.

## 3 Results and discussion

### 3.1 Letters of intent – review and disposition

A total of 87 LOIs were received, excluding resubmissions; of these, one LOI was withdrawn prior to scientific evaluation. The outcomes for the LOIs are summarized in Table 1, and the average scores and score ranges for each outcome are show, by category, in Table 2. For purposes of Table 2, any trial involving an invasive procedure, including catheterization for radiologic diagnosis and coronary artery stenting, was considered to be “surgical,” even if a pharmacologic intervention was also employed as part of study design. Five LOIs were disapproved on administrative grounds, while the rest were disapproved as consequence (at least in part) of scientific review (see Table 3).

**Table 1:** LOI outcomes by subject matter.

Category	Number Received	Approved for planning	Disapproved (Disapproved without scientific review)	“Pre-planning”	Other
Observational	6	4	2 (1)	0	0
Mental Health	16	6	6^a^ (1)	1	3^c^
Surgical	16	6	6	3	1^d^
Medical	49^f^	13	29^b^ (4)	5	3^e^
Totals	87	28	43	9	7

**One LOI was offered the opportunity for resubmission; the resubmitted LOI was approved.

a This includes one LOI offered the opportunity for revision; no revised LOI was received.

b Includes one LOI offered the opportunity to revise and resubmit. The revision was approved, but this approval is not shown in the table.

c No decision was rendered on two LOIs. The third was transferred to another funding mechanism (CCTA).

d No decision was rendered.

e No decision was rendered on two LOIs. The second was withdrawn.

f Includes one proposal including cognitive behavioral therapy for a physical condition.

**Table 2:** Average scores by LOI outcome and subject matter.

Subject Matter	Average Score (Range) for Approved LOIs	Average Score (Range) of LOIs Approved for Preplanning	Average Score (Range) for Disapproved LOIs
Observational	3.6 (3.0–4.6)	–	3.0^a^
Mental Health	4.2 (3.2–4.8)	4.0^a^	3.5 (1.8–3.8)
Medical	4.2 (3.5–4.9)	3.7 (2.7–4.3)	3.1 (1.8–4.8)
Surgical	4.2 (3.3–4.7)	4.2 (4.0–4.7)	3.6 (1.8–4.2)

a There was only a single LOI in this category.

**Table 3:** LOI outcomes by score range and subject matter.

Subject Matter	Score Range	Approved	Approved for Preplanning	Disapproved	Other
Medical	4.0–5.0	6	2	4	2
	3.0–4.0	4	2	11	–
	2.0–3.0	0	1	10	–
	1.0–2.0	0	0	1	–
Surgical	4.0–5.0	5	3	1	–
	3.0–4.0	1	–	2	–
	2.0–3.0	–	–	1	–
	1.0–2.0	–	–	1	–
Observational	4.0–5.0	1	–	–	–
	3.0–4.0	3	–	1	–
	2.0–3.0	–	–	–	–
	1.0–2.0	–	–	–	–
Mental Health	4.0–5.0	5	1	–	–
	3.0–4.0	1	–	2	3
	2.0–3.0	–	–	1	
	1.0–2.0	–	–	2	–

Three of the approved protocols were assigned to the Point of Care Research team (discussed below) for further analysis. Nine LOIs were referred for detailed discussions, referred to as preliminary planning, or “preplanning”meetings, prior to making a final decision on whether to proceed further; seven were eventually approved for further planning and two were withdrawn from consideration. Overall, 37% of all LOIs were eventually approved for planning within the traditional CSP program, as opposed to the Point of Care trials program or other VA funding mechanisms.

### 3.2 Medical and surgical clinical trial letters of intent

Most LOIs were for randomized controlled trials, consistent with the overall program scope and strength for which VA investigators are familiar. Three LOIs proposed follow-up studies on previously completed CSP trials - one was approved, one was disapproved, and one had had no action as of the time of this analysis. The approved LOI received an average score of 4.3, with no individual reviewer score below 4.0. The disapproved LOI had a score of 1.8.

During the timeframe for this analysis, CSP initiated a new effort to help with addressing fundamental challenges with clinical trials and for more rapid adoption of findings by the healthcare system. This Point of Care (POC) Research Program has been described elsewhere as one VA approach to conducting pragmatic comparative studies on existing therapies [[14], [15], [16], [17]]. Consequently, CSP received three LOIs for special consideration under this effort. Given some unique requirements for considering POC studies, these LOIs are not further discussed below (although they are shown in the “approved column” in Table 1, their scores are not included in Table 2).

#### 3.2.1 LOIs approved for planning

The lowest score leading to approval was 3.3; the highest was 4.9; eleven of 16 LOIs receiving approval for immediate planning had average scores of 4.0 or above. LOIs that were accepted for planning with scores below 4 tended to originate early in the time frame covered by this analysis. The last LOI accepted with a score in this range was submitted in November of 2013; 3 of the 4 were submitted before February of 2009. Reviewers that scored LOIs in the 4.0 and above range generally indicated that these proposals had few, if any flaws, and that the trials would have significant impact on the health care of Veterans and non-Veterans alike. Not all LOIs were disapproved as a result of reviewer scoring. Four LOIs were administratively disapproved because they were out of scope for CSP. In some cases, reviewer scores and the texts of the comments did not align; when scores seemed more positive than comments, the comments tended to have a greater influence on the final approval/disapproval decision than did the raw scores. One highly scored LOI was disapproved because, although the proposal was potentially meritorious, funding was required for other conditions more causally related to military service. Finally, one LOI, although highly regarded by reviewers and related to military service, would ultimately affect an exceedingly small number of Veterans cared for by VA; as a result, it was given a low programmatic priority. Of the 9 LOIs that were approved for preplanning, 2 were withdrawn during the preplanning process and the remaining 7 were accepted for the complete planning process.

#### 3.2.2 LOIs disapproved for planning

Disapproved LOIs tended to have substantially more flaws identified by reviewers than did approved LOIs (Table 4). Although the “Other” category included a variety of concerns, one major – and usually disqualifying – concern was that the proposed trial was not worth doing, whether because the problem was not significant, the trial was premature, or because a significant interventional advance was likely to occur prior to completion of the trial. In only rare cases did reviewers suggest that an LOI propose a trial that competed with an ongoing or planned trial addressing the same question raised in the LOI.

**Table 4:** Issues raised on scientific review of LOIs, by subject area and outcome.

Issue Area	Observational		Mental Health		Surgical		Medical		Overall	
	Approved (4)	Disapproved (1)	Approved (6)	Disapproved (5)	Approved (6)	Disapproved (6)	Approved (10)	Disapproved (26)	Approved (26)	Disapproved (38)
Investigator Qualifications	0	0	0	4	0	1	1	13	1	18
Feasibility within VA	3	0	2	2	2	2	3	8	10	12
Preliminary Data	0	1	0	3	0	3	1	19	1	26
Potential Ethical Issues	0	0	0	2	0	3	1	10	1	15
Design Concerns	0	1	0	3	3	6	6	19	9	29
Statistical Power	1	0	1	4	2	4	3	11	7	19
Competing Studies	0	0	0	0	0	1	2	0	1	2
Other	2	0	0	4	2	3	3	19	7	26

Data table includes only those LOIs that underwent scientific review.

Lack of preliminary data supporting a decision to conduct a large multisite trial was the single most important discriminator between approved and disapproved LOIs. Investigator qualification was a close second. Reviewers tended to consider lack of experience as a site investigator for a large multisite clinical trial as a significant negative; LOIs that were approved for planning often included as a co-proponent an individual who had previously played a significant role – often as study chair – in such a trial.

Issues associated with trial design were particularly common; these included lack of clarity regarding outcome measures or lack of specificity regarding the proposed intervention to lower-level concerns, such as inclusion/exclusion criteria. These concerns were sometimes characterized as potential ethical issues associated with the trial design. Issues associated with determination of sample size were frequently raised when reviewers assessed LOI submissions. While some LOIs failed entirely to include a power analysis, others relied on effect size estimates that reviewers considered to be unrealistic (and invariably too large). In a significant number of cases this left reviewers concerned that the proposed study could not be conducted within VA (although this concern was also frequent among approved LOIs).

### 3.3 Mental health clinical trials letters of intent

A high proportion of LOIs dealt with mental health, reflecting prevalent conditions among the VA patient population. LOIs covered the areas of substance abuse, depression (including suicide), PTSD, TBI, schizophrenia, and psychogenic seizures. One LOI was administratively disapproved since it was intended to study new drug for which no phase 1 or 2 studies had been performed. The 1 approved LOI with a score below 4.0 represented a comparative effectiveness study on strategies to treat a common condition (depression) to be carried out by an experienced investigator. No study with an LOI Score above 4.0 was disapproved, reflecting the high programmatic priority given to mental health investigations. In general, concerns expressed by reviewers were similar to those of medical and surgical trials.

### 3.4 Observational and genetic epidemiology study letters of intent

Six of the 87 LOIs considered in this study proposed observational investigations, covering genetics/genomics (2), deployment health (2), general Veteran health status (1), and program assessment. Four were approved for planning; 1 was administratively disapproved without outside review. Apart from genetics proposal, all were heavily focused on issues associated with military service. This, the fact that observational study LOIs were authored by very experienced investigators, and the relatively low cost of observational investigations, may account in part for the high approval rate.

Observational studies that were approved for planning received design assistance from one of four CSP epidemiology centers, which also assist in execution of the studies. Studies involving genetic epidemiology, such as those conducted using resources from the VA Million Veteran Program [18], are now evaluated using a CSSEC panel that is distinct from that used to evaluate clinical trials, but that panel was not yet in place at the time these proposals were considered.

### 3.5 Studies evaluated by the CSP Cooperative Studies Scientific Evaluation Committee

From the 87 LOIs above, 36 were ultimately accepted for full planning. This included 28 approved after initial review, 7 approved after a “pre-planning” session, and one approved after an invited resubmission. Of these 36 studies, 25 (69%) ultimately were submitted to the CSSEC for full consideration of funding. Of these 25, 22 were ultimately funded by CSP, for an ultimate success rate of 25% of all submitted LOIs, 61% of LOIs ultimately approved for planning, and 88% of fully planned proposals submitted to CSSEC.

The average score given CSSEC reviewed proposals on the first submission was 22.19, with a range of 14.5–36.7. The difference between high and low reviewer scores for these submissions averaged 10, with a range of 3–20. Ten of the studies received scores that recommended them for funding after first presentation to the CSSEC; these protocols had scores averaging 19.0 with a range of 14.3–22.4. One of the proposals approved after the first submission was ultimately not executed due to a failure to obtain matching funding.

One study was disapproved on the first submission and was not eligible for further consideration; resubmission was discouraged (though not barred and not submitted) for another; one additional study which was eligible for resubmission was never resubmitted. Twelve studies which were eligible for resubmission were ultimately approved for funding. The average score on resubmission was 18.2, with an average range among reviewers of 15.2–22.8. The difference between high and low scores for these studies averaged 8, with a range of 1–13. Both studies that were funded with scores of over 22 were of high programmatic importance to VA.

Six protocols represented submissions of high programmatic priority for VA due to relationship to combat-related mental health or physical injury. Three were funded on the first submission, and 3 after resubmission. Two of the submitted protocols represented innovative clinical trial designs, both of which were funded on first submission; they received scores of 17.5 and 21.3, respectively.

Thirty-seven CSSEC reviews (25 initial submissions and 12 resubmissions) were analyzed. Topics most frequently discussed by the CSSEC are shown in the table below, together with the number of times the issues appeared to result in a non-fundable score (based upon discussion in executive session). The most common topics brought up by members of the CSSEC were the appropriateness of the overall experimental design, including randomization and stratification, and the formulation of the primary outcome/response measure, found in 27 and 26 of the protocol discussions, respectively. The CSSEC tended to give better scores to superiority studies than to non-inferiority designs, perhaps reflecting a belief that they were more likely to significantly change clinical care. The committee scores also demonstrated a preference for objective, patient-centered outcome measures and tended to be skeptical of biochemical endpoints and subjective patient-reported responses. Considerations of study impact and generalizability were also frequently discussed (16 discussions) and were frequently a reason for a non-fundable score (6 discussions).

Studies disapproved by the CSSEC, or receiving a recommendation not to resubmit a revision, tended to have numerous defects. One proposal was judged likely to have little impact on practice; the CSSEC had difficulty understanding a clearly defined, answerable question in another.

The committee carefully examined assumptions regarding subject recruitment and event rate/effect size assumptions (17 discussions each), frequently demonstrating concern regarding the study power and the interpretation of a “negative result.”

Often an issue that concerned the CSSEC members could be dealt with in the discussion with investigators at the CSSEC meeting. As a result, many of the issues raised in the CSSEC meeting did not ultimately affect the funding decision. Sometimes the CSSEC members were convinced that their concerns were unjustified; on other occasions study investigators accepted the CSSEC recommendations and agreed to amend their proposals “on the fly.”

Analysis of the CSSEC minutes illustrated the broad range of potential concerns raised by the CSSEC members in their deliberations (Table 5), though many concerns discussed by the CSSEC were ultimately not judged to be significant or to have impact on the merit of the proposal. Not surprisingly, issues regarding study design, including determination of appropriate primary outcome measures, led the list of factors triggering discussion and the list of concerns giving rise to non-fundable scores.

**Table 5:** CSSEC discussion issues and impact on proposal outcome.

CSSEC Discussion Issue	Times Discussed	Times Affected Approval Decision
Design, including randomization and stratification	27	6
Primary endpoints/outcome measures	26	10
Secondary endpoints	19	0
Assumptions about event rate/effect size	17	4
Assumptions about subject recruitment	17	2
Study importance/generalizability	16	6
Site execution/event adjudication	15	1
Safety and adverse event reporting	14	2
Inclusion/exclusion criteria	12	1
Length of follow-up	9	1
Drug dosing	9	1
Blinding	9	0
Bias	9	0
Power analysis	6	3
Missing data	6	1
Will results be made obsolete by practice changes?	6	0
Cost-effectiveness analyses	2	1

#### 3.5.1 *Publication outcomes of* funded *CSP studies*

Because of the long period required for execution, analysis, and publication of clinical trials results, there is limited data on publications resulting from the work considered above; most of the 22

Funded studies are ongoing. For studies that have given rise to publications other than “design papers,” the publications on study outcomes are found in Table 6. Other papers are in preparation or in various stages of the submission and review process but have not been published.

**Table 6:** Studies Included in this review for which the primary paper has been published.

Study Number	Title	LOI Submission Date	Funding Approval Date	Primary Publication Date	Number of publications	Primary publication journal (year)	References	Citations
517-FS	Randomized On/Off Bypass Follow-up Study (ROOBY-FS)	4/6/10	4/15/13	8/17/17	5	NEJM*	[[33], [34], [35], [36], [37], [38], [39], [40], [41]]	188
576	VA Augmentation and Switching Treatments for Improving Depression Outcomes (VAST-D)	7/4/08	10/29/10	7/11/17	5	JAMA	[[42], [43], [44], [45], [46], [47], [48], [49]]	76
578	Prevention of Serious Adverse Events Following Angiography	11/24/08	7/20/11	11/12/17	2	NEJM	[[50], [51], [52], [53]]	307
588	Randomized Endo-vein Graft Prospective - REGROUP - Trial	10/14/10	2/11/13	1/10/19	3	NEJM	[[54], [55], [56], [57]]	49
589	Veterans Individual Placement and Support Towards Advancing Recovery (VIP-STAR)	11/24/10	1/3/13	4/1/18	4	JAMA Psychiatry	[[58], [59], [60], [61]]	25

†Citations are counted only for the primary outcome paper, taken from Google Scholar on 4/20/2021.

*New England Journal of Medicine.

## 4 Conclusions

In a scientific peer review process, clinical trial applications require several elements to give reviewers and the funder/sponsor confidence on the investment and potential impact of the study. Within VA, a two-step process involving reviews at an LOI and full proposal stage may allow some insights into factors that contribute to success or not. LOIs require the same level of thought and effort that go into any successful application for funding. While these submissions covered a range of disease areas and topics, several common themes arose that highlight areas of emphasis among reviewers. Successful proponents effectively demonstrate that the problem which they are trying to address is important to the Veteran community, but also that it is of national importance and is likely to be of general medical interest. LOIs focusing on economic issues tended to fare poorly by comparison with those proposing studies focused on achieving optimal medical outcomes. “Successful “LOIs (those that were approved for planning) left no doubt as to primary question or outcome measure and included sample size estimates based upon careful power analyses using a clinically meaningful effect size supported by preliminary data from the investigator or from the literature. LOIs containing overestimates of likely effect size (as perceived by reviewers), tended to be viewed very negatively. Similarly, unsupported estimates of the number of potential subjects (or the fraction of potential subjects likely to enroll) proved likely to convince reviewers that a proposed trial is not actually likely to succeed in VA.

Approved LOIs demonstrated awareness of ongoing or planned investigations which might be perceived as “competing,” and demonstrated clearly why the proposed study was complementary, not redundant. Given challenges of recruitment [[19], [20], [21]], the decision to fund a proposal similar to an ongoing trial requires clearing a high bar. The successful LOIs were supported by an abundance of preliminary data which clearly established that there was a significant clinical controversy which needed to be resolved, or a significant problem for which sufficient evidence to generate practice guidance was not available. Preliminary data cited in successful LOIs clearly demonstrated that the proposed investigation could be successfully executed within VA, showing realistic estimates of the number of Veterans who would meet inclusion/exclusion criteria within a reasonable number of clinical trial sites. Vague statements based upon clinical experience were not helpful; hard data was necessary to support a strong LOI.

Not surprisingly, successful LOIs were most commonly generated by investigators who provided convincing evidence that they are likely to successfully execute a complex multisite clinical investigation. Such efforts extend beyond clinical/scientific expertise. Within VA, the clinical leader on CSP proposals is referred to as a study chair to imply their role in managing a diverse group of experts required to complete study requirements. Investigators who have served as Co-PI with a more experienced investigator, who served as site investigators on multisite trials, and who served as principal investigators on several single-site trials were more likely to be viewed favorably than those who did not demonstrate this level of experience. Importantly, success as a basic science investigator was not viewed positively in the absence of clinical trials experience.

The high rate of approval for studies ultimately accepted for planning seems likely to result from two factors. The first of these is rigorous scientific review of the initial LOI. Simply put, bad ideas, poor designs, and unqualified investigators are unlikely to get past this “gatekeeping” step. The second is the formal CSP trial planning process which involves not only those who initially propose a study, but also a team of statisticians, regulatory affairs, pharmacy, and clinical trials support personnel who are able to refine proposals, assure the use of appropriate statistical methodology, and (following trial initiation) assure that the trial is conducted according to rigorous standards. Even with these processes in place, only 10 of 25 proposals submitted to CSSEC received a “fundable score” during the initial proposal review.

Although our analysis of clinical trial funding application success and failure is not directly applicable outside the VA, it seems likely that similar considerations will apply to other organizations that use LOIs; potential investigators should prepare them with no less care than they give to full research proposals. It seems likely that the kinds of issues frequently raised during CSSEC proposal evaluation will be the types of concerns important to other scientific review committees. Many of the review criteria proposed for clinical trials by NIH were routinely evaluated by VA in both the LOI and final review processes for clinical trials, though there were some differences. Study importance, design, and timeline, together with investigator qualifications, are included in the NIH review criteria and were, as noted above, thoroughly critiqued in all phases of the VA review process. The environment for research, one of the NIH criteria, was not a major consideration for CSP proposals. This may reflect CSP's use of permanent statistical and pharmacy coordinating centers to support trial management, data collection, and analysis. Innovation in trial design, while welcomed when appropriate, was not a major independent factor in CSP LOI or proposal evaluations, perhaps because design innovations are most commonly appropriate for early phase trials that are not considered appropriate for CSP.

PCORI expands upon NIH review criteria with 65 distinct methodology standards falling into 16 topic areas. For the most part these standards deal with data integrity and data analysis – topics important in NIH evaluations and equally important in CSP evaluations of LOI's and full proposals. Although PCORI's emphasis on patient-centeredness was occasionally echoed in the CSP process, in general CSP evaluations followed a more traditional medical model.

Our focus in conducting this programmatic review was strictly on examining the factors affecting success and failure in a funding process focused solely on clinical investigations; we have not attempted to address issue of gender or other bias in peer review [22,23], the ability of the peer review process to foster innovation or discriminate among proposals likely to have more or less scientific impact [[24], [25], [26]], the format by which applications should be reviewed and discussed prior to funding [27,28], or the reproducibility of the peer review process [5,29,30]. Our observations on the factors influencing success in the application process are similar to those that have been observed in applications submitted for funding to the Plastic Surgery Foundation [31]. Issues of significance and overlap of the proposed research played an important role in review of initial LOIs for CSP projects, but usually were not of concern to the CSSEC – perhaps because the LOI process resolved them prior to submission of a full application. Issues associated with study design and execution, dominated CSSEC discussions. These results are consistent with those identified in a study of factors influencing NIH funding which, since it considered both clinical and nonclinical research, had a somewhat less granular analysis [32].

Given the similarities in NIH, PCORI, and CSP review criteria, it is possible that through consideration of the issues relating to success and failure of LOIs and full proposals submitted to the VA that investigators both within and outside of VA may prepare more effective applications, reducing the time associated with resubmissions and shortening the time between the conception of an important research study and the ultimate publication of its results. Based on CSP experience, investigators should pay particular importance to creating clear and understandable study designs in which both the scientific question and the primary outcome measure are clinically significant and easily understood. This outcome measure, which is particularly likely to be a matter of discussion during evaluation of both LOIs and final proposals, should be patient-centered, clinically meaningful, and reproducibly measured in a manner that can be implemented in everyday medical practice.

## Declaration of competing interest

The authors declare that they have no known competing financial interests or personal relationships that could have appeared to influence the work reported in this paper.
