# Supplementary material for: Clinical trials proposed for the VA Cooperative Studies Program: Success rates and factors impacting approval
Source: Contemp Clin Trials Commun. 2021 Jul 9;23:100811. doi: 10.1016/j.conctc.2021.100811 (PMC8287148; doi:10.1016/j.conctc.2021.100811)
Supplement: Multimedia component 1 [file mmc1.docx]

**Supplemental Appendix**

**Part 1 – Preparation of Letters of Intent (taken from** [**https://www.research.va.gov/programs/csp/update/guide.pdf**](https://www.research.va.gov/programs/csp/update/guide.pdf)**, last accessed 07/06/2021)**

The submission of a Letter of Intent (LOI) by an eligible VA clinician-investigator to CSPCO is the first step in CSP study-development process. The individual submitting the LOI is designated as the Principal Proponent. The LOI is a detailed proposal that describes the research question, need for a cooperative study, and ideas for how a CSP study may potentially achieve study objectives. An LOI should be no longer than 10 pages and contain the following information:

**Title & Principal Proponent Name(s).** A Co-Principal Proponent may be named when a clear and justifiable need exists. No more than two Co-Principal Proponents may be named without prior CSPCO approval.

**Objectives** of the proposed research, including concise mention of patients/participants, records or biospecimens, any interventions or interactions, and outcomes.

**Relevance** and potential impact of the study to VA and to Veterans and how the study will affect clinical practice. Statements on any clinical equipoise, expected implementation of findings, and/or incorporation into practice guidelines are strongly encouraged.

**Feasibility** **and justification** for conducting a multisite or large-scale observational study within VA.

**Summary of the preliminary research** that has been accomplished with data to support a largescale investigation.

**Proposed study design** that includes the following items as appropriate:

- - Study population, with specific inclusion and exclusion criteria
  - Interventions, interactions, or treatments and services to be compared
  - Outcomes or endpoints to be evaluated
  - Research design (randomized trial, observational cohort study) and rationale
  - Sampling strategy
  - Logical links among questions, data, and primary and secondary associations
  - Number of participants, records, or biospecimens, and number of participating VAMCs o Duration of study
  - Data sharing plan
  - Resources required (Full-Time Employees [FTEs], Full-Time Equivalent Employees [FTEEs], and estimated total costs)
  - Methods of data collection
  - Units of measurements, strategies for analyses
  - Other details, as needed

**Acknowledgment** of VA policy to include women and minorities in research and adherence to CSP policies overall.

If desired, include a statement on experience and/or qualifications for conducting a multisite or largescale observational study.

The following documents also should be included in the submission, but do not count toward the 10-page limit:

**Completed Form 10-1313-13** or equivalent. (See https://www.va.gov/vaforms/medical/pdf/10-1313- 13-fill.pdf .)

**Statement of disclosure**. A formal statement that confirms the absence of a financial or contractual relationship between the Principal Proponent and any proposed organization involved in the trial that may constitute a real or apparent conflict of interest. If such a relationship or contract does exist, or appears to exist, the Principal Proponent must provide full disclosure.

**Statement of eligibility**. To be eligible for planning support, the Principal Proponent must either have at least a 5/8 VA appointment or have applied for and received a 5/8 appointment waiver from the Director, CSP, within the previous year approving an LOI submission. In the latter case, a copy of the waiver approval establishing eligibility to receive funds should be attached to the request. A Principal Proponent may not be a VA Central Office employee.

**Cover letter** from the Principal Proponent’s VAMC Director and the Associate Chief of Staff for Research and Development (ACOS/R&D) acknowledging and approving the submission and time to dedicate to required activities.

**Curriculum Vitae (CV)** of the Principal Proponent(s) with VA address, email, telephone, and fax numbers (NIH Biosketch is acceptable if it provides sufficient expertise and experience information).

**Potential Planning Committee members**. Names, addresses, telephone numbers, and email addresses of five to seven experts who would be appropriate for the study Planning Committee should the LOI be approved. The list should include potential VA SIs.

**List of ongoing and submitted proposals** that are directly related (e.g., pilot study, single-site, and smaller clinical trial) to the study proposed in the LOI and the funding source.

**Suggested subject matter experts (SMEs).** A list of names and affiliations of SMEs who could serve as potential reviewers, Data Monitoring Committee (DMC) members, or other roles not directly related to study planning or conduct may be included on a separate page.

**Part 2 – Evaluation of Letters of Intent (instructions to reviewers)**

In reviewing the enclosed proposal, please keep in mind that this is a request for planning support. This is the initial review in the planning process. If planning support is approved, the study will be assigned to one of the Cooperative Studies Program Centers. The principal investigator and a biostatistician at the Center, together with the study’s Planning Committee, will develop the final protocol. The Cooperative Studies Scientific Evaluation Committee will then review the proposed research plan. Details such as specific inclusion/exclusion criteria, specific treatment regimens, follow-up techniques, data collection forms, sample size estimation, data processing procedures and statistical analyses need not be included in the investigator’s proposal at this stage.

Please comment on the following:

1. DESCRIPTION

Briefly describe the proposed study and the specific objectives.

2. CRITIQUE

1. What is your general impression of the importance and timeliness of this study, especially to the VA population?
2. Are the objectives and the endpoints clearly stated?
3. Are there logical links between questions, data and endpoints?
4. Are there sufficient preliminary data from smaller studies to
   justify a large scale multicenter clinical trial/epidemiological study?
5. Is the population of subjects to be studied (or the unit of
   analysis) clear and appropriate?
6. Is the estimation of the number of available study patients and
   sampling strategy reasonable?
7. Are the proposed methods, “state of the art”?
8. Are there any ethical problems that may jeopardize the study
   plan?
9. Are the drugs/devices or services to be studied likely to be
   supplanted by newer drugs/technology or services prior to the
   completion of the study?

3. INVESTIGATOR QUALIFICATIONS

1. Is the investigator trained and experienced in the types of
   procedures/intervention proposed?
2. Is the investigator knowledgeable about the field of research
   proposed?
3. What is the investigator’s record of achievement in scientific research?
4. Is investigator qualified to lead/direct a national multi-site clinical trial/epidemiological study?

**NOTE:** It is helpful, but not required, for the investigator to have experience in cooperative studies. However, the scientific and administrative support of the CSP Coordinating Center can potentially help compensate for an investigator’s lack of experienc

1. RECOMMENDATIONS

Keeping in mind that this is a request to plan a cooperative study, please indicate your overall assessment by typing your rating in the space provided using the scale below as a guide.

A score in the mid-range indicates a judgement that the study should be planned, but with specified modifications. Please add any additional comments.

**RATING: ______**

_

1 2 3 4 5

Reject Approve

Additional comments:
